# Sex Determination via the Second Cervical Vertebra and Odontoid Process: A Case Report and a Review of the Literature

**DOI:** 10.3390/diagnostics14131446

**Published:** 2024-07-06

**Authors:** Emanuela Stan, Camelia-Oana Muresan, Raluca Dumache, Veronica Ciocan, Stefania Ungureanu, Ecaterina Daescu, Alexandra Enache

**Affiliations:** 1 Department of Neuroscience, Discipline of Forensic Medicine, Bioethics, Deontology and Medical Law, “Victor Babes” University of Medicine and Pharmacy, 300041 Timisoara, Romania; emanuela.stan@umft.ro (E.S.); raluca.dumache@umft.ro (R.D.); veronica.luta@umft.ro (V.C.); stefania.ungureanu@umft.ro (S.U.); enache.alexandra@umft.ro (A.E.); 2 Institute of Legal Medicine, 300610 Timisoara, Romania; daescu.ecaterina@umft.ro; 3 Ethics and Human Identification Research Center, Department of Neurosciences, “Victor Babes” University of Medicine and Pharmacy, 300041 Timisoara, Romania; 4 Department I of Anatomy and Embryology, “Victor Babes” University of Medicine and Pharmacy, 300041 Timisoara, Romania

**Keywords:** skeletal remains identification, odontoid process, sex estimation, the second cervical vertebra

## Abstract

Determining an individual’s sex is crucial in several fields, such as forensic anthropology, archaeology, and medicine. Accurate sex estimation, alongside the estimation of age at death, stature, and ancestry, is of paramount importance for creating a biological profile. This profile helps narrow the potential pool of missing persons and aids identification. Our research focuses on the second cervical vertebra and odontoid process, which is particularly valuable due to their high sexual dimorphism. This brief research is structured as follows: we provide an overview of morphometric analysis of the second cervical vertebra for accurate sex estimation in forensic anthropology. We then delve into a case report to explore sexual dimorphism of the C2 vertebrae. Moreover, we discuss some of these studies that showed a significant correlation between the dimensions of the second cervical vertebrae and height, suggesting that the C2 can be used as a reliable indicator for stature estimation. The high accuracy rate of sex estimation using the second cervical vertebrae suggests that this method is a valuable tool for forensic anthropologists. Its practical application can significantly contribute to identifying and profiling individuals in a forensic context, thereby aiding in the identification process.

## 1. Introduction

Forensic anthropology comprises a range of interdisciplinary efforts, incorporating comprehensive anatomical expertise to facilitate accurate human identification [1]. To ascertain an individual’s identity, anthropological examinations and medico-legal investigations involving human remains rely on the investigator establishing a biological profile. Accurate identification is of utmost importance in forensic investigations. Misidentification can have serious consequences, including the wrongful conviction of an innocent individual or the failure to provide closure for a victim’s family. A multidisciplinary approach to identification is crucial to minimize these risks and ensure justice is served. By combining various methods, forensic investigators can increase the chances of identification and provide accurate information about the victim. Research and case studies have shown that a multidisciplinary approach to identification is highly effective. For example, a study conducted by C.C. Radu et al. [2] combining methods such as anthropological examination, facial reconstruction, post-mortem computed tomography, and DNA analysis resulted in a success rate for positive identification. The multidisciplinary approach to identification involves several steps. The first step is an anthropological examination, which provides information about the individual’s biological profile. This includes factors such as age, sex, and ancestry. The next step is facial reconstruction, which can be performed using graphic reconstruction and sculptural portraits made of clay. This process aims to create a visual representation of the victim, which can aid identification. In addition, post-mortem computed tomography is used despite not being a commonly used method in current forensic practice in Romania. This technique improves the results of medico-legal investigations by providing detailed images of the body. Finally, DNA analysis confirms the decedent’s identity [2].

Determining sex in forensic anthropology mainly relies on examining distinctive skeletal characteristics that differ between males and females, such as the morphology of the pelvis and skull. Accurately determining sex is crucial in forensic anthropology as it plays a significant role in establishing a biological profile by estimating skeletal age and stature. Precise estimation of sex can substantially aid in narrowing down potential matches when identifying human remains in criminal investigations or mass disasters. Moreover, determining sex and age is also crucial in cases involving living individuals, such as crimes involving minors, child pornography, and the influx of migrants without valid identification documents [3,4].

Previous studies have shown that the second cervical vertebra exhibits enough sexual dimorphism in terms of its size to accurately determine an individual’s sex using morphometric analysis [5,6,7,8]. The C2 vertebra possesses unique morphological features that make it easy to identify and distinguish from other vertebrae. Additionally, research has revealed that cervical vertebrae, including C2, are better preserved than other vertebra [7]. These findings suggest that analyzing the size and morphology of the C2 vertebra can provide reliable information about an individual’s sex, making it an ideal candidate for sex determination in forensic anthropology. The high preservation rate of cervical vertebrae also makes them valuable for sex determination in archaeological and forensic contexts. However, one major drawback of these methods is that they rely on the preservation of several bones or vulnerable skeletal elements which are often missing or damaged in forensic investigations. In such cases, forensic anthropologists have to rely on smaller bones to estimate age, sex, and height. Examining smaller bones can provide valuable information because they are more likely to be preserved than long bones [9]. Additionally, analyzing smaller bones allows for a more comprehensive analysis of skeletal remains when long bones are not available or are fragmented. Therefore, ongoing research and development of advanced techniques are imperative in providing more precise and objective results in these sensitive situations. Techniques such as genetic markers or digital imaging analysis could provide more reliable and objective results in these complex scenarios [10,11,12].

This study aims to give an overview of morphometric analysis of the second cervical vertebra for accurately determining sex in forensic anthropology. Additionally, we will examine a case report to explore the sexual differences in the C2 vertebrae compared to traditional anthropological examination.

## 2. Case Presentation

In September 2023, police discovered the body of an unidentified person caught in several branches fallen into the water on the left side of a river in Timis County. The body was found about 3 m away from the shore and towards the middle of the river.

### 2.1. Autopsy Findings and Anthropological Examination

For identification purposes, the biological profile of the individual was drawn up, examining the skeletal remains to define race, sex, age, and height.

From the evaluation of the skull’s anthropomorphological characteristics, it was concluded that the corpse belonged to a Caucasian person.

#### 2.1.1. Sex Estimation

**Figure 1:**
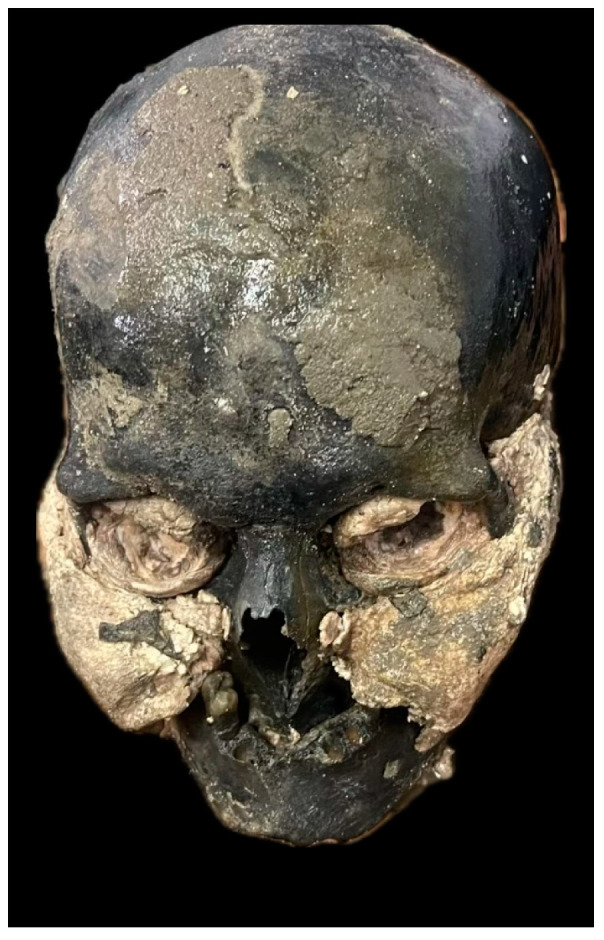
Cranium, male.

1. The medial surface of the ischiopubic branch forms a wide and flat ridge; ventral arc slight ridge; convex subpubic concavity; ischiopubic ramus ridge is broad and flat [13,14].
2. The large sciatic incision is narrow in phase 4 (specific to the male sex) [15].
3. Massive nuchal crest with marked bone projection (stage 5); voluminous male-specific mastoid process (stage 4); supraorbital edge thick, rounded (stage 4); glabella prominent, massive (stage 4) [16]. Figure 1.

Through the study of the skull and pelvis, it was established that the corpse was a male subject.

#### 2.1.2. Age Estimation

Several anthropological methods, based on the observation of particular bone elements, were used to define the approximate age at death of the individual.

**Figure 2:**
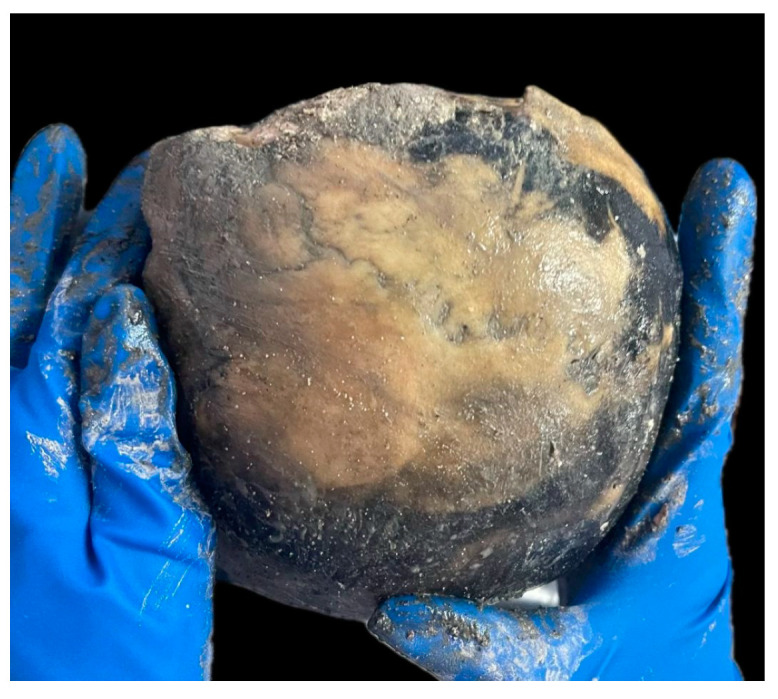
Cranial sutures, completely obliterated.

**Figure 3:**
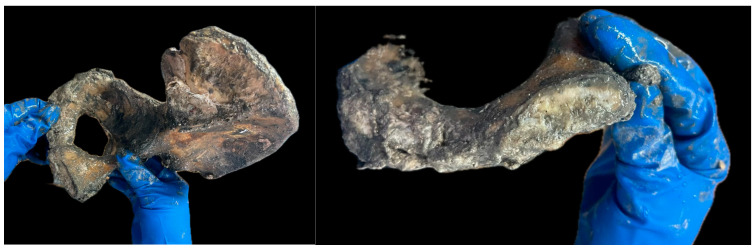
Male pelvis.

1. Completely obliterated cranial sutures: 3rd degree of evolution [17]. Figure 2.
2. Sternal extremity of ribs: porous recess, edge with sharp protrusions [18].
3. Ventral margin of pubic symphysis eroded with excavated surface [19]; symphyseal face with depression [20]; auricular surface with marginal lipping, macroporosity, absence of transverse organization with increased irregularity [21]. Figure 3.

The data showed an advanced age above 65 years.

#### 2.1.3. Stature Estimation

Finally, the calculation of the approximate stature was based on the measurement of the length of long bones. Karl Pearson’s formula [22] for males was used to estimate stature from the femur (81.306 + 1.88 × femur length = 165 cm) and from the tibia (78.664 + 2.376 × tibial length = 159 cm). This allowed us to conclude that the corpse had a height of about 159–165 cm.

#### 2.1.4. Autopsy Conclusions

The body belongs to a male person, over 65 years old, with a waist of about 159–165 cm, in an advanced state of decomposition, being skeletonized in a proportion of 90%. The advanced state of putrefaction and lack of soft tissues do not allow us to determine the type of death and the cause of death. No traumatic injuries were detected on the remaining bone fragments. The corroboration of all the data from the autopsy, with the police investigation, under the limits due to the state of conservation of the body, indicated that the death occurred at least 4–6 months before the date of discovery of the body.

### 2.2. Sex and Height Estimation from the Second Cervical Vertebra

**Figure 4:**
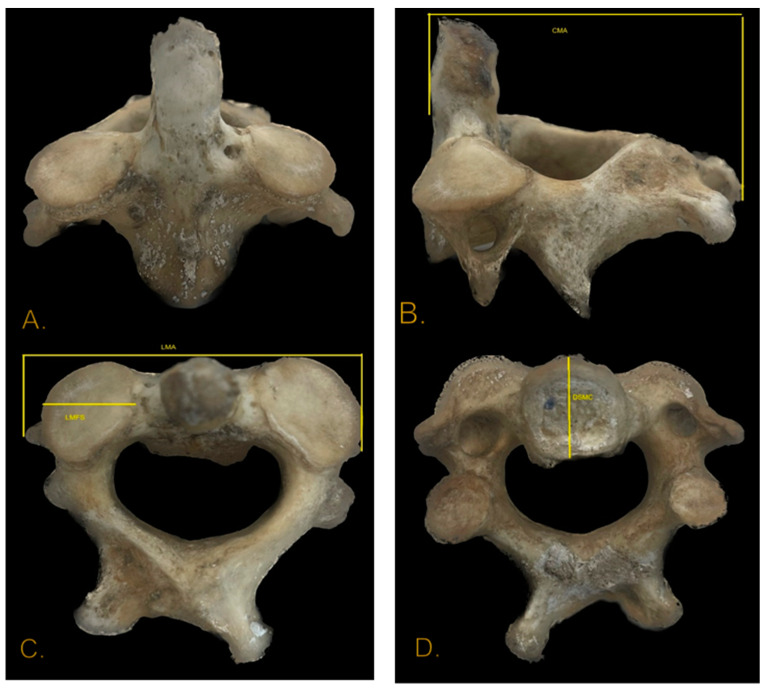
Case from the archive of Institute of Forensic Medicine Timisoara. Measurements of the axis. (**A**) Anterior view. (**B**) Lateral view: CMA (maximum length of the axis). (**C**) Superior view: LMA (maximum width of the axis), LMFS (maximum width of the superior facet). (**D**) Inferior view: DSMC (sagital maximum body diameter).

By examining the skull and pelvis, we were able to estimate the age and sex of the individual. We then compared our measurements (Figure 4) of the second cervical vertebra with the results obtained by Gama et al. [7] in a study conducted on a European population. The original study, conducted by Gama et al. [7], described a logistic regression model using the most predictive variables (LMA, DSMC, CMA, and LMFSD) to determine the age and sex of a European population. So, to ensure the robustness of our findings and to observe if our results are comparable with other European populations, we meticulously introduced our values in the logistic regression model described by Gama et al. according to the following equation: L = −62.170 + (0.561 × CMA) + (0.677 × LMA) + (−0.818 × LMFSD) + (0.977 × DSMC). Our measurements for males of LMA (54.4), DSMC (15.8), CMA (55.6), and LMFSD (18) were not only comparable with the values obtained for the CISC sample and the ISC-XXI sample from the original study but slightly higher for all parameters. Negative values are associated with female individuals, whereas positive values correspond to male individuals. In our case, the value of L was 8.563, which is consistent with a male individual compared to the data obtained from other available bones.

This study attempted to estimate stature using the formula developed by Rodriguez et al. [23]. In anthropological studies, linear regression formulas are commonly used to estimate stature based on different skeletal measurements. Rodriguez et al. [23] developed these formulas using tomographic imagery of the first and second cervical vertebrae on a sample of the contemporary Spanish population. They considered the height of the vertebra (V) and the interforaminal length (I) for the first cervical vertebra (C1) and the greatest diameter of the dens (DO) and the height of the odontoid (O) for the second cervical vertebra, as these measurements showed the highest correlation coefficient with height. The regression formula developed for males was used to estimate stature in this study: S = 72.47 + 0.92O + 1.28DO + 0.33V + 0.56I [23]. The result was slightly higher (173.75) than those obtained from other long bones (166). It is important to note that this formula was developed specifically for males in the Spanish population. Therefore, when applied to other populations or individuals, it may have limitations or variations. However, given the lack of available formulas specifically developed for different populations, using this formula for males provides a reasonable estimation of stature in this case. Moreover, it is worth noting that the measurements in this case were taken from fresh vertebrae, whereas the original study by Rodriguez et al. [23] used CT images. When using dried vertebrae, measurement variations may occur due to shrinkage or deformation of the bone structure over time. In our case, the vertebrae were fresh, without deformation due to time lapse, but the absence of soft tissue may affect the accuracy of certain measurements. These differences between fresh vertebrae and CT image measurements could explain the slightly different results.

## 3. Discussion

### 3.1. Sex Estimation Based on the Second Cervical Vertebra

The cervical vertebrae have a thick outer layer of bone called the cortical layer, and their small surface areas are less exposed to environmental factors that can affect bones. Furthermore, the cervical vertebrae have unique physical features that set them apart from other bones in the body. For instance, transverse foramina and bifid spinous processes are absent in any other skeletal element. These distinctive features make it easier for forensic anthropologists to identify cervical vertebrae accurately while analyzing human remains [6,24].

The atlas (C1) and axis (C2) are the most useful cervical vertebrae for determining sex. Males tend to have larger muscle mass, bone density, and brain weight than females. As a result, the atlas has a stronger link with the skull base and can sustain more weight in males than in females. This leads to more prominence in the upper and lower articular faces and the area of the vertebral foramen in males. During the onset of puberty, bone elements undergo significant sexual dimorphism due to the influence of estrogen and testosterone. This hormonal influence causes the development of secondary sexual characteristics such as increased bone density and changes in bone shape. These changes are particularly noticeable in the axis dens, which contributes to overall sexual dimorphism in adults. Furthermore, males’ increased physical power and muscle mass considerably affects bone landmarks [5,6,25,26].

In a study on sexual dimorphism of the second cervical vertebra, Wescott [5] proposed a quantitative method of determining the sex of adult individuals. The study involved 100 males and 100 females selected from the Hamann–Todd and Terry anatomical collections, which included a wide age range of 20 to 79 years. Wescott utilized digital sliding calipers to measure eight dimensions of the C2 vertebra (Table 1), with accuracy rates ranging between 81.7% and 83.4%. The results of the study indicated statistically significant differences between males and females, suggesting that sexual dimorphism exists in the second cervical vertebra. These findings could impact the understanding of biomechanical differences between genders.

The method developed by Wescott was tested by Marlow and Pastor [6] on a sample of 153 individuals from the Spitalfields’ anatomical collections held at the National History Museum, London. In addition to the eight measurements described by Wescott [5], the authors included an extra measurement of C2 (width of vertebral foramen). They discovered that the maximum sagittal length and the maximum amplitude between the upper articular faces of the axis were the most significant discriminatory values for analyzing sexual dimorphism. The results of their analysis showed a range of valid categorization percentages between 70.91% and 78.9%. The discriminant function analysis revealed an accuracy rate of 83.3% in classifying individuals as male or female. This percentage was higher than the classification accuracy achieved by Wescott [5]. These categorization percentages demonstrate the effectiveness of Wescott’s method in precisely classifying individuals from the Spitalfields anatomical collection. The results suggest that the method is reliable and can be used confidently in similar datasets.

Bethard and Seet [8] conducted a study in a contemporary American population sample to evaluate the effectiveness of Westcott’s method [5] in accurately classifying individuals. The researchers achieved a success rate of up to 86.7%, indicating that the method proposed by Westcott [5] shows promising results in classifying individuals within the current American population.

In another study conducted by Gama et al. [7], the researchers aimed to determine the accuracy of using measurements of the second cervical vertebra to determine the sex of individuals. The study involved 190 individuals from the Coimbra Identified Skeletal Collection and 47 individuals from the Identified Skeletal Collection of the 21st Century (ISC-XXI). The researchers measured 13 dimensions of the second cervical vertebra following Westcott’s method. The study found that the predictive model showed a high level of accuracy, ranging from 86.7% to 89.7%, in correctly identifying the known sex of the individuals in the sample. The metrics that exhibited the most discriminatory ability were the maximum width of the axis (LMA), sagittal maximum body diameter (DSMC), the maximum length of the axis (CMA), and the maximum width of the right superior facet (LMFSD).

A recent study conducted by Rozendaal et al. [24] aimed to develop and validate sex estimation functions based on cervical vertebrae measurements. The study examined a sample of 1020 vertebrae from 295 adult individuals of European ancestry, whose remains were part of the Athens collection at the University of Athens human skeletal reference collection in Greece and the Luis Lopes skeletal collection in Lisbon, Portugal. The age range of the individuals included in the study ranged from 20 to 99 years. The researchers used maximum body height (CHT), vertebral foramen anterior–posterior diameter (CAP), and vertebral foramen transverse diameter (CTR) to develop discriminant functions from the seven cervical vertebrae. The findings of this research demonstrate that only CHT and CTR displayed statistically significant sexual dimorphism, with accuracy rates ranging from 80.3% to 84.5%. The study found no significant distinctions between the contemporary Greek and historic Portuguese skeletal samples with respect to demographic characteristics. Thus, the discriminant functions used for estimating sex based on cervical vertebrae may not be influenced by specific populations or time periods [24]. These results contribute to improving forensic and bioarchaeological investigations by providing reliable methods for sex estimation from cervical vertebrae in individuals of European ancestry within a wide age range. However, it is important to note that further research is needed to validate these findings and explore potential variations in sex estimation based on cervical vertebrae in other populations or historical contexts.

A study conducted by Torimitsu et al. [27] aimed to assess the accuracy of sex determination by analyzing nine measurements obtained from the second cervical vertebra of 244 deceased individuals through post-mortem computed tomographic images within a Japanese population. The researchers discovered that the maximum distance between superior articular facets (DMFS) and maximum width of the axis (LMA) showed significant sexual dimorphism, with expected cross-validated accuracies of 83.5% and 83.1%, respectively. These findings suggest that the measurements of the second cervical vertebra, especially DMFS and LMA, can serve as reliable indicators for determining sex in the Japanese population. The high accuracy rates further emphasize the potential of post-mortem computed tomographic images as a non-invasive method for forensic investigations related to sex assessment.

In their study, Xu et al. [28] aimed to determine the importance of the morphology of the second cervical vertebra and the posterior projection of the C2 pedicle axis in surgical intervention. The study analyzed 50 s cervical vertebrae from individuals aged between 21 and 68 years, calculating 18 linear and four angular parameters for both male and female specimens. The study found significant differences in the morphological measurements between males and females. The variables with significant sex differences were the anterior and posterior height and diameter of the vertebral body. For males, the mean anterior height was 21.1 ± 1.7 mm, and for females, it was 19.5 ± 1.7 mm. The mean posterior height was 16.5 ± 1.6 mm for males and 15.3 ± 1.1 mm for females. In regards to the dens, only the diameter exhibited a statistically significant difference between males (with an average diameter of 10.3 ± 0.7 mm) and females (with an average diameter of 9.6 ± 0.9 mm). The dimensions of the pedicle, breadth, height, and length also showed significant size differences between the sexes.

The second cervical vertebra (C2), commonly referred to as the axis, is frequently utilized in forensic anthropology for sex determination. This preference stems from its well-defined morphological characteristics and its notable preservation even under harsh environmental conditions. Numerous studies have explored various methods and measurements of the C2 to enhance the accuracy of sex determination. The following table (Table 2) summarizes the literature evidence discussed in the context of these studies, highlighting the citations, study focus, methods performed, and characteristics of each study.

### 3.2. Height Estimation Based on the Second Cervical Vertebra

The correlation between individuals’ heights and the morphology of their spinal vertebrae is a prominent determinant in forensic medicine for identification. Forensic experts can estimate a person’s height with a high degree of accuracy by analyzing the size, shape, and alignment of the vertebrae. This information can be crucial in identifying or matching unknown remains to missing persons’ records. Furthermore, understanding the relationship between spinal morphology and height can help reconstruct the overall physical profile of an individual, which can provide valuable insights for criminal investigations or archaeological studies. Examining each component of the spinal cord, especially in the cervical region, can facilitate the identification of height in cases where height assessment is challenging, such as in cases of severe trauma or highly fragmented bodies [29,30]. Many studies have explored the links between the size of the second cervical vertebra and stature in different populations. These studies have produced differing results, indicating that factors such as genetics, nutrition, and overall health may impact the connection between the second cervical vertebra and height [23,31,32]. Mostafavi [31] conducted a study to determine if specific linear dimensions of the second cervical vertebrae can predict the height of the adult population in Iran. The study used a three-dimensional computed tomography scan (3D CT scan) to measure 15 indexes of the second cervical vertebrae in 66 individuals (33 males and 33 females) who were at least 18 years old. The study’s analysis investigated the relationship between height and the second cervical vertebrae indexes. The 15 indexes measured were as follows: the maximum height of the axis (AMA), the maximum length of the axis (CMA), the odontoid process sagittal diameter (DSD), the odontoid process transverse diameter (DTD), the maximum distance between the superior facets (DMFS), the maximum length of the superior facet (CMFS), the maximum width of the superior facet (LMFS), the length of the vertebral foramen (CMFV), the sagittal maximum body diameter (DSMC), the maximum width of the vertebral foramen (LMFV), the maximum height of the odontoid process (AMD), the maximum transverse diameter of the body (DTMC), the maximum width of the axis (LMA), the maximum length of the inferior facet (CMFI), and the maximum width of the inferior facet (LMFI). The results showed that only three indexes, AMA (r = 0.470, *p* = 0.0001), CMA (r = 0.320, *p* = 0.007), and DSMC (r = 0.281, *p* = 0.019), had a significant positive correlation with height. According to the study, there is a significant link between the size of the second cervical vertebrae and height in the adult population of Iran. Therefore, these measurements are a dependable way of estimating height in this group [31]. However, more research is required to verify these results and determine whether they apply to other populations beyond Iran.

Torimitsu et al. [32] conducted a study that analyzed the correlations between stature and measurements of the second cervical vertebra in the Japanese population. The study used multidetector computed tomography to gather data from 216 Japanese subjects, comprising 116 males and 100 females. The researchers found a positive correlation between height and all measurements of the C2. These measurements include the distances from the top of the dens to the anteroinferior point of the vertebral body (DA), the top of the dens to the posterior point of the spinous process (DS), and the anteroinferior point of the vertebral body to the posterior point of the spinous process (AS). The study also revealed that the length of the second cervical vertebra strongly correlates with height. Furthermore, DA was found to have the highest correlation coefficient (r = 0.762), while AS had the lowest (r = 0.684). This suggests that the anteroinferior point of the vertebral body (DA) can be a reliable indicator for height estimation in the Japanese population.

A study conducted by Rodríguez et al. [23] focused on assessing height using the first and second cervical vertebrae among a population from Spain. The researchers collected data from tomographic images taken with a dental CT of 203 healthy individuals from a Spanish population aged between 15 and 84. The best correlation was observed while analyzing individuals of unknown sex, where four measurements were considered. Specifically, the measurements were acquired from the first cervical vertebra (height of vertebra (V) + interforaminal length (I)) and two from the second vertebra (greatest-diameter dens (DO) + height of the odontoid (O)). The regression formula, depending on the sex of the individual, was as follows: S = 75.62 + 0.85O + 1.19DO + 0.41V + 0.43I (for females) and S = 72.47 + 0.92O + 1.28DO + 0.33V + 0.56I (for males). This regression formula allowed for accurate sex determination in cases where the sex was unknown, regardless of whether the individual was male, female, or part of the entire population. The researchers concluded that measuring the height of the vertebrae, interforaminal length, greatest-diameter dens, and height of the odontoid provided the most reliable results. These findings suggest that the combination of these four measurements can accurately determine the sex of an individual, regardless of their unknown sex. This regression formula provides a reliable method for forensic anthropologists to identify the sex of skeletal remains.

Genetic analysis represents a powerful tool in forensic identification and sex determination due to its high accuracy and ability to provide conclusive results even in cases where morphological characteristics are ambiguous or degraded [33]. However, integrating or excluding genetic analysis in the diagnostic algorithm depends on several factors.

Preservation and condition of remains:

Morphological Identification: The second cervical vertebra (C2) often remains well-preserved even under harsh conditions, making it a reliable source for morphological analysis. Its unique features offer distinctive markers for sex determination.

Genetic Analysis: It is important to note that genetic material may be compromised in cases where skeletal remains are significantly degraded, posing a challenge to DNA analysis and potentially making it unfeasible [10].

Resources:

Morphological Identification: This method is less resource-intensive and can be performed with standard forensic anthropological tools and expertise. It is a viable option in many forensic contexts, particularly when resources are limited.

Integrated Approach: Combining morphological and genetic analyses can significantly enhance the reliability of sex determination and overall identification processes. Morphological methods can provide immediate insights, while genetic analysis can confirm findings, particularly in complex or disputed cases [11]. This comprehensive approach is a valuable tool in forensic practice.

Exclusion of Genetic Analysis: In cases where the morphological characteristics of the C2 are sufficiently distinct and the preservation of the vertebra is excellent, genetic analysis may be deemed unnecessary. This approach conserves resources and expedites the diagnostic process.

Inclusion of Genetic Analysis: When morphological markers are inconclusive or the skeletal remains are incomplete, integrating genetic analysis becomes critical for accurate sex determination and identification.

In conclusion, the integration or exclusion of genetic analysis in the diagnostic algorithm should be guided by the condition of the remains, available resources, time constraints, and the specific requirements of each forensic case. While morphological analysis of the second cervical vertebra offers a robust method for sex determination, the inclusion of genetic analysis can provide an additional layer of accuracy and certainty, especially in challenging cases. Thus, a flexible approach that considers the strengths and limitations of both methods is recommended for optimal forensic practice.

## 4. Conclusions

It can be concluded that the second cervical vertebrae display significant sexual differences and offer a high rate of accuracy for determining sex. Moreover, using cervical vertebrae for this purpose is particularly helpful in cases where other skeletal components are missing or poorly preserved. The high level of accuracy in determining sex using the second cervical vertebrae indicates that this method is a valuable tool for forensic anthropologists and can contribute to identifying and characterizing individuals in a forensic setting.

## Figures and Tables

**Table 1:** The dimensions measured in the Wescot study [5].

Maximum Sagittal Length (XSL)	The sagittal length of the vertebra from the most anterior point on the body to the posterior edge of the spinous process.
Maximum Height of the Dens (XDH)	The height from the most inferior edge of the anterior border of the body to the most superior point on the dens.
Dens Sagittal Diameter (DSD)	The maximum sagittal (anteroposterior) diameter of the dens.
Dens Transverse Diameter (DTD)	The diameter of the dens measured perpendicular to the sagittal diameter.
Length of Vertebral Foramen (LVF)	The internal length of the vertebral foramen measured at the inferior edge of the foramen in the median plane.
Maximum Breadth Across the Superior Facets (SFB)	The maximum breadth between the superior articular facets as measured from the most lateral edges of the superior facets.
Superior Facet Sagittal Diameter (SFS)	The maximum sagittal diameter of the superior articular facet.
Superior Facet Transverse Diameter (SFT)	The maximum transverse diameter of the superior articular facet measured perpendicular to the sagittal diameter.

**Table 2:** C2 analysis for sex determination: summary of literature evidence.

	Study Focus	Methods Performed	Characteristics of the Study
Wescott (2000) [5]	Quantitative method for sex determination using C2	Digital sliding calipers to measure eight dimensions of C2 (as described in Table 1)	100 males and 100 females from Hamann–Todd and Terry anatomical collections, ages 20–79; statistically significant differences between males and females, accuracy: 81.7–83.4%
Marlow and Pastor (2011) [6]	Testing Wescott’s method on a different sample	Added an extra measurement (width of vertebral foramen); discriminant function analysis	153 individuals from Spitalfields’ anatomical collections; most significant discriminatory values: the maximum sagittal length and the maximum amplitude between the upper articular faces of the axis; a range of valid categorization percentages between 70.91% and 78.9%., discriminant function analysis accuracy rate of 83.3%
Bethard and Seet (2013) [8]	Evaluating Wescott’s method in a contemporary American sample	Applied Wescott’s method	Contemporary American population sample; accuracy: up to 86.7%
Gama et al. (2015) [7]	Accuracy of using C2 measurements for sex determination	Measured 13 dimensions following Wescott’s method	190 individuals from Coimbra Identified Skeletal Collection and 47 from ISC-XXI; the predictive model showed a high level of accuracy, ranging from 86.7% to 89.7%
Rozendaal et al. (2020) [24]	Developing and validating sex estimation functions based on cervical vertebrae	Maximum body height, vertebral foramen anterior–posterior and transverse diameter	1020 vertebrae from 295 adults of European ancestry (Athens and Luis Lopes skeletal collections); accuracy: 80.3–84.5%
Torimitsu et al. (2016) [27]	Assessing accuracy of sex determination using C2 in a Japanese population	Nine measurements obtained from post-mortem CT images	244 deceased individuals; significant measures: DMFS, LMA; accuracy: 83.5–83.1%
Xu et al. (1995) [28]	Importance of C2 morphology in surgical intervention	Analyzed 18 linear and 4 angular parameters	50 s cervical vertebrae from individuals aged 21–68; significant sex differences in morphological measurements

## Data Availability

Data are contained within this article.
